# Emotional Assessment in Spanish Youths With Antisocial Behavior

**DOI:** 10.3389/fpsyg.2021.671851

**Published:** 2021-05-25

**Authors:** Juan García-García, María José Gil-Fenoy, María Blasa Sánchez-Barrera, Leticia de la Fuente-Sánchez, Elena Ortega-Campos, Flor Zaldívar-Basurto, Encarna Carmona-Samper

**Affiliations:** ^1^Department of Psychology, Center for Health Research, University of Almería, Almería, Spain; ^2^Department of Personality, Evaluation and Psychological Treatment, University of Granada, Granada, Spain

**Keywords:** antisocial behavior [APA PSYNET], emotional assessment, executive functions hot, IAPS, juvenile justice, younger offenders

## Abstract

Impaired emotional capacity in antisocial populations is a well-known reality. Taking the dimensional approach to the study of emotion, emotions are perceived as a disposition to action; they emerge from arousal of the appetitive or aversive system, and result in subjective, behavioral, and physiological responses that are modulated by the dimensions of valence, arousal, and dominance. This study uses the International Affective Picture System (IAPS) to study the interaction between the type of picture presented (pleasant, neutral, or unpleasant) and group (adolescents under custody in juvenile justice centers, adolescents under non-custodial measures, and secondary school students) in the emotional assessment of these dimensions. The interaction between the study variables was statistically significant. Statistically significant differences were found between the three types of pictures presented, in the ratings of unpleasant pictures between the custody group and the group of secondary students in regular schooling in valence, and in the ratings of unpleasant, neutral, and pleasant pictures in arousal, between the custody group and all groups. Discriminant analyses of each affective dimension indicate that the unpleasant pictures with violent and/or aggressive content tend to be in the functions that most differentiate the antisocial groups.

## Introduction

Scientific evidence agrees in pointing to impaired emotional capacities as a fundamental characteristic of habitual offenders or people who tend toward antisocial behavior (e.g., Farrington, 1989; Muñoz-García et al., 2003; Garaigordobil et al., 2004; Alcázar-Córcoles et al., 2010). Emotional processing, in turn, is related to higher cognitive capacities or hot executive functions. In this way, executive functions (EF) have an important role in development and may therefore be relevant in the appearance of frequent risk behaviors in adolescence, such as antisocial behavior, and more specifically, behaviors relating to violation of criminal law (Gil-Fenoy et al., 2018). EFs have been related mainly to the exercise of different cognitive processes, however, they are also involved in certain emotional responses, behaviors, and expressions. The executive processes and cortical networks associated with EF functioning suggest the existence of two differentiated circuits: the executive control circuit (dorso-lateral), involved in cold, more cognitive EFs, and the socio-emotional circuit (orbito-frontal and ventro-medial), involved in hot processes related to behavioral and emotional control (Zelazo et al., 2004; Zelazo, 2020). Incorporating this assumption, Steinberg (2008) proposes a risk-taking model in adolescent development that is characterized by a dual system, greater risk-taking in adolescence can be understood in terms of asynchronous changes in the maturation of a “cognitive control” system responsible for self-regulation processes and a socio-emotional system of “incentive processing,” responsible for processes related to reward, emotion, and social cognition. More specifically, Botdorf et al. (2017) suggested that individual differences in cognitive control can predict risk taking, but only when control is assessed in conditions of greater affective arousal, indicating that researchers who work with cognitive control in the laboratory must use tasks that evoke an affective state in order to adequately explain risk decision making in adolescence.

Consequently, alteration or dysfunction in the hot network, which is more sensitive to social and emotional stimuli, can cause emotional, interpersonal, or social problems such as high reactivity, self-regulation problems, affective lability, problems in processing and awareness of emotions, and problems in behavior-directed control of emotions. In fact, it has been noted that adolescents with better EFs process emotional information better, leading them to more flexible adaptation to their environment and less risk of psychopathology (Morea and Calvete, 2020). In this way, impaired emotional processing (lack of empathy, emotional lability, difficulty recognizing emotions, etc.) is characteristic of habitual offenders or subjects who tend toward antisocial behavior and psychopathy (e.g., Muñoz, 2004; Moreno et al., 2009; Halty-Barrutieta and Prieto-Ursúa, 2015; Pincham et al., 2015).

The assessment of emotions, therefore, takes on a special role in this context, and the use of a dimensional approach to emotion, such as Lang's biphasic model, can offer an adequate evaluative framework. Lang (1995) defines emotions as action dispositions that facilitate adaptation to the environment and are triggered by significant stimuli. He describes affective space from two dimensions: affective valence and arousal, although other authors have noted a third dimension of dominance, closely correlated to valence (Osgood et al., 1957). According to Lang's theory, emotions arise from the arousal of one of two motivational systems, the appetitive or the aversive, according to the valence dimension of the stimulus (Lang, 2010). Emotions regulate the somatic and autonomic responses (LeDoux, 2000; Davis and Lang, 2003), producing cortical, autonomic, and behavioral changes that vary in their intensity in the arousal dimension (Cacioppo and Berntson, 1994). A majority of studies, in all populations, tend to present a boomerang effect in the two-dimensional emotional space defined by these two dimensions, in other words, very pleasant and very unpleasant stimuli tend to produce much arousal, while stimuli with neutral valence are related to less arousal (Moltó et al., 1999, 2013; Bradley and Lang, 2000, 2007; Vila et al., 2001).

The International Affective Picture System (IAPS) was developed from this theoretical foundation. The instrument has proven to be reliable and stable, since pictures with similar content occupy close positions in the two-dimensional space (Patrick and Lavoro, 1997), and various physiological responses have been related both to assessed valence (startle reflex, heart rate, and electromyographic activity) and to arousal (electrical conductance of the skin). Thus, the IAPS has become one of the most widely used standardized instruments in experimental research for emotional assessment with pictures (Moltó et al., 2013). In Spain, different adaptation studies for the adult population have been carried out (Moltó et al., 1999, 2013; Vila et al., 2001), and the IAPS has been used to study how different emotional stimuli influence other cognitive processes such as memory (Fernández-Rey and Redondo, 2007; Gordillo-León et al., 2010), learning (López et al., 2009; Redondo and Méndez, 2011), and attention (García and Calvo, 2010), as well as its use in the field of addictions (Aguilar et al., 2008, 2011), distress (Zangróniz et al., 2017), and eating and weight disorders (Miccoli et al., 2014, 2018).

Regarding use of the IAPS with antisocial samples, certain studies in an adult population have focused on the alteration of certain physiological responses (Serafim et al., 2009). Studies focusing on emotional assessment of the pictures are less frequent. Pastor et al. (2003) worked in this line with adult psychopaths, not finding statistically significant differences in emotional assessments of the affective dimensions. They interpreted this fact as an emotional deficit that is covered by expressive–evaluative language appropriate to the emotions, typical of psychopathy.

However, the adult psychopath population presents characteristics that differ from the adolescent antisocial population. Previous studies have demonstrated that antisocial youths with callous and unemotional traits (CU) are impaired in processing negative or even positive emotional stimuli (Truedsson et al., 2019), and there is much evidence to support a link between emotion processing and antisocial behavior that appears before adulthood (Pincham et al., 2015). This study seeks to compile evidence along this line from a dimensional approach to emotion.

The main aim of the present study, therefore, is to examine the emotional assessment of Spanish youths with sanctionable antisocial behavior, using the set of IAPS pictures corresponding to their age range, and to analyze the influence of the interaction of two variables: the type of picture/emotional stimulus presented (pleasant, neutral, and unpleasant) and group membership (subjects under custodial or non-custodial sanctions or in normal schooling). In addition, like a second objective, we expect to obtain validity evidences for the use of IAPS in Spanish adolescents.

## Materials and Methods

### Participants

The sample was composed of 160 youths divided into three groups according to the degree of antisocial behavior they presented: two antisocial groups, one with youths in custody with judicial sanctions (*n* = 59; 66.1% male) and other, youths subject to non-custodial measures with judicial sanctions (*n* = 40; 72% male), and a comparison group of secondary students in normal schooling (*n* = 61; 68.9% male). All groups were from Andalusia (Spain), with ages between 14 and 21 years, with an average age of 16.68 (SD = 1.54), and 68.8% of the sample being male. The sample was selected by intentional sampling, with all participants being volunteers. It was controlled that there were neither statistically significant differences in average age (*F*_welch_ = 1,708, *p* = 0.18), nor in the percentage of men within each group (χ^2^ = 0.320; *p* = 0.88).

### Instruments

In order to measure the adolescents' emotional assessment, we used a set of 58 pictures (descripts in **Table 4**), targeted for children and adolescents, from the IAPS—International Affective Picture System (Lang et al., 2008). This instrument was designed to be a set of standardized stimuli, differentiated in their affective dimensions, which would situate the subject in a specific affective space. A response notebook containing 58 manikins of the Self-Assessment Manikin Scale (SAM; Bradley and Lang, 1994) was used to measure the adolescents' emotional assessments by the three dimensions of valence, arousal, and dominance prompted by each picture. The notebook was 10 pages long, with six manikins per page (four on the last page), each manikin preceded by the number of the picture to be assessed. For each of the affective dimensions, SAM has a scale of five human pictograms that are ordered according to the intensity of the dimension. For each picture, participants were to indicate one of the pictograms, or one of the spaces between them, for each dimension (row); in this way they assigned the level of valence, arousal, and dominance that they felt with each picture. Scoring for the pictograms ranges from 1 (least pleasing, least arousal, and least control) to 9 (most pleasing, most arousal, and most control). The three SAM dimensions served as dependent measures.

### Procedure

Permission was first obtained from the competent authorities in juvenile justice and education systems in Andalusia (Spain). The study objectives were presented to the official authorities and to the school principals, and to the parents/guardians of the adolescents. Whenever the youths were underage, parental authorization was obtained. The assessment session was carried out on an individual basis, in all cases meeting the requirements of legislation on personal data. The study was approved by the University Bioethics Commission of Research with Humans, University of Almeria (ref. 170214). The participation of all the young people in the study was voluntary.

A PowerPoint presentation was used to present the pictures on a laptop computer. The location of the assessment session in each study group was chosen for maximum adequacy within the possibilities of each institution. Location requirements were as follows: silence, adequate lighting for distinguishing the pictures, and a comfortable desk and chair for working.

Before beginning the assessment of the 58 pictures, the adolescents were instructed in how to use the SAM template, and three pictures were presented as practice: one pleasant, one neutral, and one unpleasant. After checking that the adolescents had properly understood how to respond and rate the pictures, we proceeded to present the 58 pictures for assessment. Prior to seeing each picture, the participants were first presented with a preparation slide (“Get ready to rate the next slide”) that was presented for 5 s. Then, the picture to be rated was presented for 6 s, after the picture left the screen, the subject indicated their ratings of pleasure, arousal, and dominance using SAM. Three to four different picture orders were used, which balanced the position of a particular exemplar within the entire series of pictures.

### Data Analyses

A mixed factorial ANOVA 3 × 3 (group × picture) was conducted to analyze the existence of statistically significant differences in the affective dimensions (valence, arousal, and dominance) according to the variables group (in custody, under non-custodial measures, and secondary students) and type of picture (pleasant, unpleasant, and neutral).

The statistical significance was set at *p* < 0.05, being the effect sizes estimates for the significant effects reported by $\eta_{\text{p}}^{2}$ for the MANOVA and ANOVA results and non-standardized means differences for *post-hoc* pairwise comparisons. When statistically significant interaction effects emerged, the simple-effects analysis procedure was performed by *post-hoc* pairwise comparisons, between and within groups, using Bonferroni corrected *t*-tests. The assumptions of the ANOVA models and *t*-tests were assessing previously on each set of data, when some non-compliance occurred, it is reported in the results, indicating the alternative test used in such cases.

In addition, a discriminant analysis was carried out for each of the affective dimensions (valence, arousal, and dominance), using group for the grouping variable (in custody, non-custodial measures, and secondary students).

All the statistical analyses were performed using IBM's SPSS Statistics 26 program (IBM Corporation, 2019).

## Results

The assumptions analysis for the mixed 3 × 3 (group × picture) ANOVA showed the non-compliance of sphericity for the three dimensions, assessed by Mauchly's test. Therefore, the multivariate tests were used to estimate the effects. The results indicated a statistically significant effect of the group × picture interaction {Λ_Wilks_ = 0.79, [*F*_(12, 304)_ = 3.13], *p* < 0.001, η${}_{\text{p}}^{2}$ = 0.11}, indicating significant differences in the three dimensions of emotion taken together across types of pictures, because of the specific group. To specify this interaction effect, simple-effects analysis was executed for the three dimensions separately. Table 1 shows the results of the pairwise comparisons between type of picture within each group, reflecting statistically significant mean differences in practically all comparisons in the three affective dimensions. The pleasant pictures received the highest scores in the dimensions of valence and dominance, followed by the neutral pictures, and finally the unpleasant ones. In the arousal dimension, scores were ordered inversely, that is, the unpleasant pictures obtained a higher score and the pleasant pictures had lower scores.

**Table 1:** *Post-hoc* pairwise comparison according to type of picture.

**Dimension**	**Group**	**Picture**		**Means Difference (SE)**	**95% CI**
Valence	Custody	Pleasant	Unpleasant	5.066^*^ (0.224)	[4.525, 5.607]
			Neutral	0.353 (0.178)	[−0.078, 0.783]
		Unpleasant	Neutral	−4.713 (0.299)	[−5.436, −3.990]
	Non-custodial	Pleasant	Unpleasant	5.843^*^ (0.272)	[5.186, 6.500]
			Neutral	0.477 (0.216)	[−0.046, 1.000]
		Unpleasant	Neutral	−5.366^*^ (0.363)	[−6.244, −4.488]
	Student	Pleasant	Unpleasant	5.870^*^ (0.220)	[5.337, 6.402]
			Neutral	0.484^*^ (0.175)	[0.061, 0.908]
		Unpleasant	Neutral	−5.385^*^ (0.294)	[−6.096, −4.674]
Arousal	Custody	Pleasant	Unpleasant	−2.216^*^ (0.340)	[−3.039, −1.394]
			Neutral	−0.529^*^ (0.152)	[−0.897, −0.162]
		Unpleasant	Neutral	1.687^*^ (0.395)	[0.731, 2.644]
	Non-custodial	Pleasant	Unpleasant	−3.858^*^ (0.413)	[−4.856, −2.859]
			Neutral	−0.433 (0.184)	[−0.879, 0.013]
		Unpleasant	Neutral	3.425^*^ (0.480)	[2.263, 4.587]
	Student	Pleasant	Unpleasant	−4.150^*^ (0.334)	[−4.958, −3.341]
			Neutral	−0.725^*^ (0.149)	[−1.086, −0.364]
		Unpleasant	Neutral	3.425^*^ (0.389)	[2.484, 4.366]
Dominance	Custody	Pleasant	Unpleasant	2.228^*^ (0.236)	[1.657, 2.800]
			Neutral	−0.473^*^ (0.160)	[−0.859, −0.087]
		Unpleasant	Neutral	−2.701^*^ (0.283)	[−3.385, −2.017]
	Non-custodial	Pleasant	Unpleasant	3.060^*^ (0.287)	[2.365, 3.754]
			Neutral	−0.640^*^ (0.194)	[−1.109, −0.171]
		Unpleasant	Neutral	−3.700^*^ (0.343)	[−4.531, −2.869]
	Student	Pleasant	Unpleasant	2.685^*^ (0.232)	[2.123, 3.247]
			Neutral	−0.523^*^ (0.157)	[−0.903, −0.144]
		Unpleasant	Neutral	−3.209^*^ (0.278)	[−3.881, −2.536]

* *p <0.05*.

On the other hand, the between-groups comparisons of each group in the picture types showed statistically significant differences only in the dimensions of valence and arousal. Specifically, in the valence dimension, we observed statistically significant differences only in the unpleasant pictures between the custody and comparison group, showing that the custody group presented higher scores, that is, they perceived these pictures as more pleasant than the comparison group. With respect to the arousal dimension, the results indicated statistically significant differences between the custody group and the other two groups in the three types of pictures. Concretely, the custody group presented higher scores, that is, more activation, than the comparison group and non-custodial group for the pleasant and neutral pictures, but showed an inverse patron of differences for the unpleasant pictures, in which the custody group showed fewer scores that the other two groups, indicating less activation to unpleasant stimuli. These results are shown in Table 2.

**Table 2:** *Post-hoc* pairwise comparisons according to group.

**Dimension**	**Picture**	**Group**		**Mean Difference (SE)**	**95% CI**
Valence	Unpleasant	Custody	Student	0.645^*^ (0.217)	[0.119, 1.172]
Arousal	Pleasant	Custody	Non-custodial	0.772^*^ (0.260)	[0.142, 1.402]
			Student	1.159^*^ (0.232)	[0.597, 1.721]
	Unpleasant	Custody	Non-custodial	−0.869^*^ (0.327)	[−1.659, −0.079]
			Student	−0.774^*^ (0.291)	[−1.479, −0.070]
	Neutral	Custody	Non-custodial	0.869^*^ (0.344)	[0.037, 1.700]
			Student	0.964^*^ (0.306)	[0.222, 1.705]

* *p <0.05*.

Regarding the discriminant analysis for each of the affective dimensions, results indicated the existence of two functions in each dimension: for valence, function 1 (F1) accounts for 70.8% of the variance (canonical *R*^2^ = 0.64) and function 2 (F2) accounts for 29.2% (canonical *R*^2^ = 0.42); in arousal, F1 accounts for 67.6% of the variance (canonical *R*^2^ = 0.60) and F2 for 32.4% (canonical *R*^2^= 0.42); and in dominance, F1 covers 63.7% of the variance (canonical *R*^2^ = 0.60) and F2, 36.3% (canonical *R*^2^ = 0.46). Moreover, Wilks' lambda indicates that the interaction between the two functions differentiates between the study groups in all dimensions: valence Λ_Wilks_ = 0.20, χ^2^ (116) = 203.86, *p* < 0.001; arousal Λ_Wilks_ = 0.22, χ^2^ (116) = 185.06, p <0.001; dominance Λ_Wilks_ = 0.21, χ^2^ (116) = 200.62, *p* < 0.001. Table 3 presents the centroids of functions 1 and 2 for each group and in each of the affective dimensions. Table 4 shows the F1 structure for each affective dimension.

**Table 3:** Functions with group centroids.

**Group**	**Valence**		**Arousal**		**Dominance**	
	**F1**	**F2**	**F1**	**F2**	**F1**	**F2**
In custody	1.65	−0.34	1.28	0.63	−1.49	−0.44
Non-custodial measures	−0.33	1.46	0.30	−1.43	0.18	1.60
Comparison	−1.40	−0.63	−1.54	0.34	1.32	−0.62

**Table 4:** Structure of function 1 for each dimension.

**Code and description**		**V**	**A**	**D**	**Code and description**		**V**	**A**	**D**
1040	Snake	0.06	0.02	−0.13^*^	5950	Lightning	0.05	**–**0.00	**–**0.02
**1120**	**Snake**	**0.03**	**−0.05**	−0.20^*^	**6230**	**Aimed gun**	0.33^*^	−0.27^*^	−0.30^*^
**1280**	**Rat**	**0.01**	0.06^*^	**0.04**	**6300**	**Knife**	0.35^*^	−0.26^*^	−0.25^*^
**1300**	**Pit Bull**	0.27^*^	**−0.09**	−0.12^*^	**6370**	**Masked man**	0.33^*^	−0.29^*^	−0.36^*^
1710	Puppies	−0.06	0.07^*^	0.01^*^	7000	Rolling pin	0.01	0.01	−0.00
1750	2 Bunnies	−0.07	0.12^*^	0.00	7010	Basket	0.02	0.14^*^	0.12
1920	Dolphins	0.14^*^	0.16^*^	0.05^*^	7030	Iron	0.04	0.10^*^	0.09
1930	Shark	0.05	−0.07	−0.07	7040	Dustpan	−0.05	0.12	0.16
2070	Baby	0.02	0.16^*^	0.01	7080	Fork	−0.02	0.1	0.06
2120	Angry man	−0.01	−0.08^*^	−0.11^*^	7090	Book	−0.09	−0.18^*^	0.13^*^
2130	Angry woman	0.01	0.00	−0.06	7100	Fire hydrant	−0.09	0.19^*^	0.13
2190	Neutral man	−0.08	0.06	−0.04	7130	Truck	−0.11^*^	0.15^*^	−0.02
2280	Neutral boy	0.03	0.06	0.05	7150	Umbrella	−0.13^*^	0.19^*^	0.14
2320	Girl reading	−0.04	0.17^*^	0.06	7170	Light bulb	−0.061	0.10	0.06
2650	Boy/ice cream	−0.02	0.07	−0.03	7250	B–day cake	−0.03	0.10^*^	0.07^*^
2660	Baby/sink	0.02	0.18^*^	0.02^*^	7330	Ice cream	−0.05	0.11	0.02
2780	Actor/Makeup	0.04	0.04	−0.08	**7380**	**Roach/Pizza**	**0.00**	0.12^*^	**0.04**
2650	Boy screaming	0.13^*^	−0.04^*^	−0.03^*^	7390	Popsicles	−0.06	0.13^*^	0.01
2890	Twins	0.16^*^	0.03	−0.14^*^	7400	Candy	−0.07^*^	0.15^*^	0.09^*^
2920	Clown	−0.13^*^	0.17	0.06	7410	M and M	−0.06	0.19^*^	0.02^*^
**3230**	**AIDS patient**	**0.03**	**−0.01**	**−0.03**	7430	Snickers	0.01^*^	0.14	0.02
3280	Boy/dentist	0.07	−0.04	−0.1	7510	Skyscraper	−0.06	0.12	−0.06
**3500**	**Man/subway**	0.35^*^	−0.20^*^	−0.26^*^	8260	Motorcycle	0.05^*^	0.03	−0.00
**3530**	**Man/Arrested**	0.28^*^	−0.22^*^	−0.28^*^	8490	Rollercoaster	−0.00	−0.06	−0.03^*^
5020	Flowers	−0.05^*^	0.10^*^	0.06^*^	8510	Sports car	0.10	0.08	−0.05
5030	Flowers	−0.04^*^	0.11^*^	0.06^*^	8620	Circus horse	0.02	0.02	−0.08^*^
5450	Space shuttle	−0.13^*^	0.15^*^	0.05^*^	**9050**	**Plane crash**	**0.07**	**−0.06**	**−0.00**
5480	Fireworks	−0.08	0.10^*^	−0.07^*^	**9421**	**Soldier**	0.04^*^	**−0.02**	**−0.03**
5910	Fireworks	−0.09	0.15^*^	−0.00	**9480**	**Skulls**	**0.05**	0.04^*^	**−0.03**

* *p <0.05. V, valence; A, arousal; D, dominance*.

**Figure 1:**
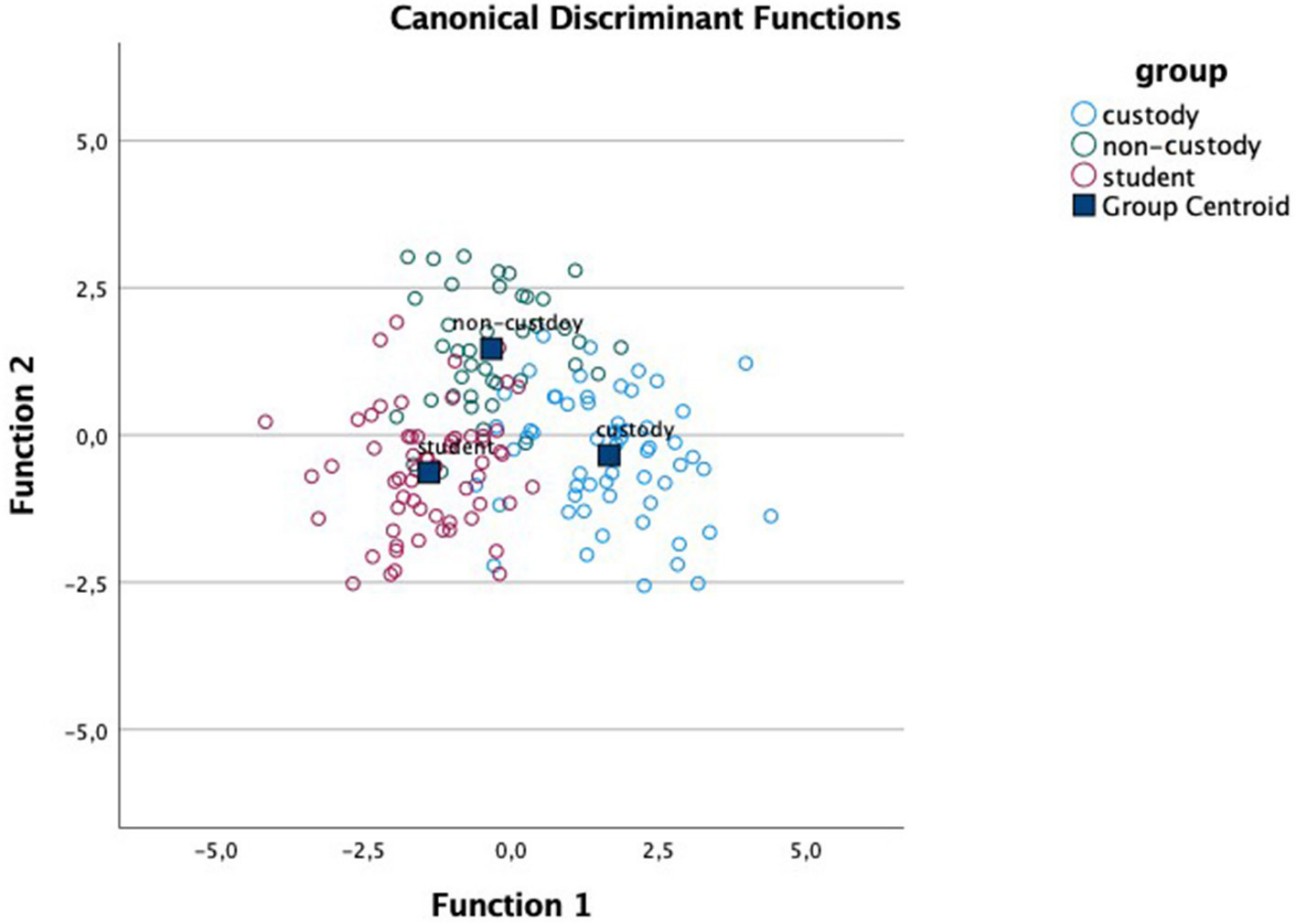
Canonical discriminant functions for the dimension of valence.

**Figure 2:**
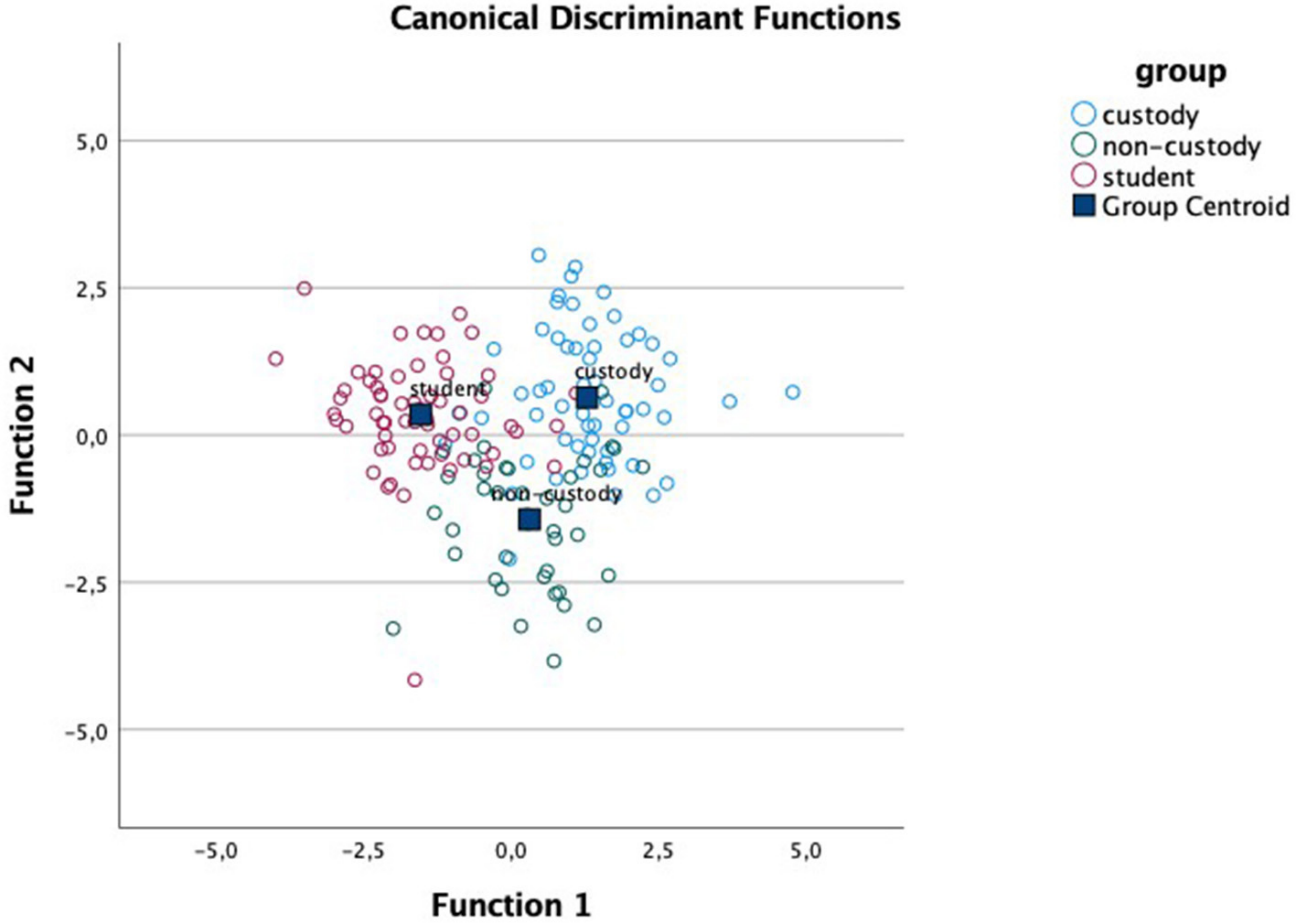
Canonical discriminant functions for the dimension of arousal.

**Figure 3:**
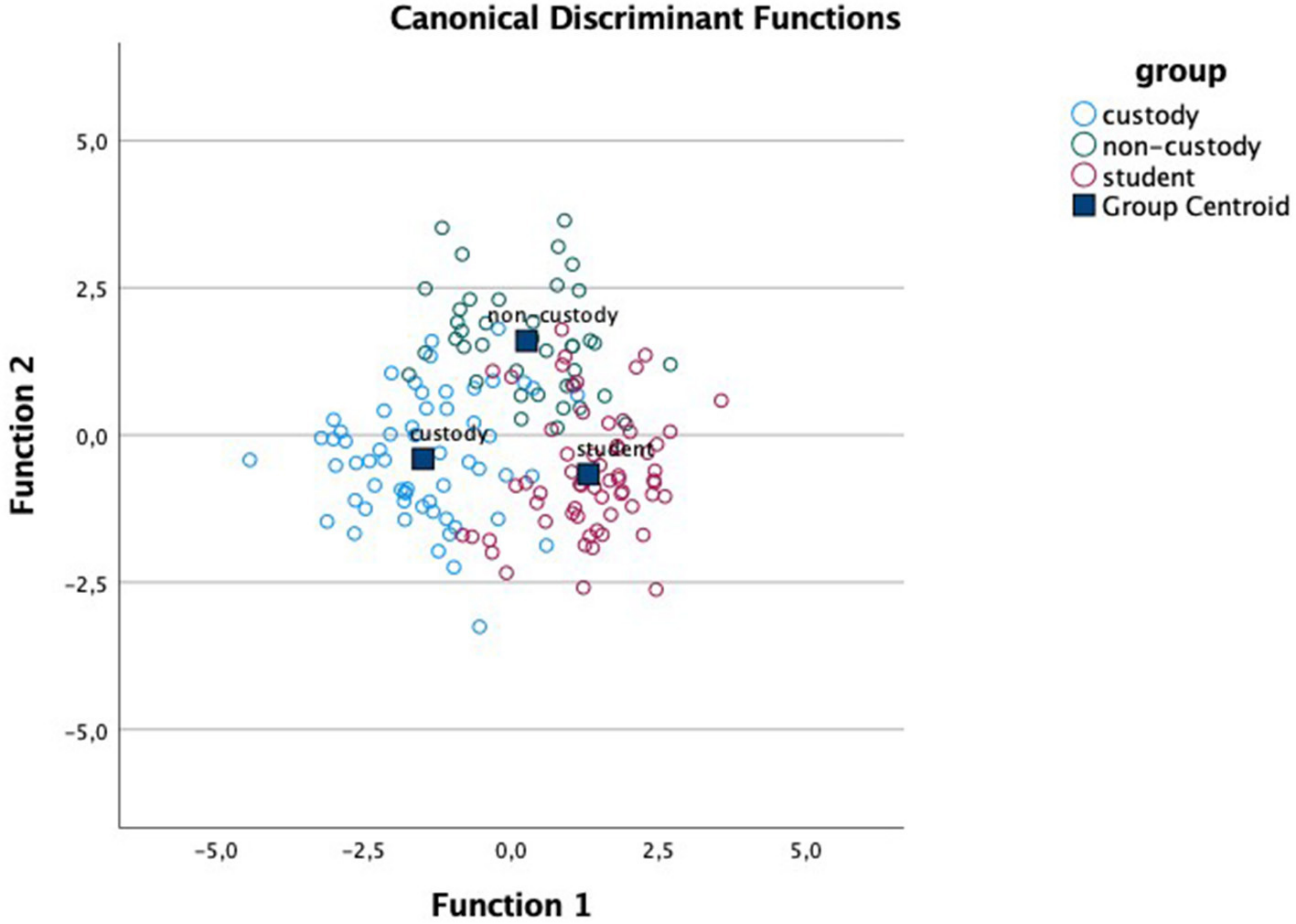
Canonical discriminant functions for the dimension of dominance.

Given that the ANOVA results showed statistically significant differences mainly in the unpleasant pictures, we focus our analysis on these. For ease of location, Table 4 shows these pictures and their data in bold type. As we can see, the unpleasant pictures whose content is violent and/or aggressive (3500, 3530, 6230, 6300, and 6370) are the only pictures that load on F1 in all the affective dimensions. This function is associated with a greater proportion of variance and is the function that most differentiates between the comparison group and the antisocial groups as seen in Figures 1–3.

## Discussion

We highlight the following aspects based on the results obtained from analyzing the influence of picture type and group on the emotional assessments of the set of pictures.

First, the statistically significant differences found according to picture type provide evidence of the discriminate validity and adequacy of the IAPS set of pictures for a Spanish population in this age range, in line with scores in US rankings. Statistically significant differences were found in all dimensions and between all the study groups; hence, regardless of the characteristics relating to antisocial behavior in part of the sample, the stimuli fulfill their function of differentiating the processing of pleasant, neutral, and unpleasant emotions. Despite these results, the Spanish adaptation of this picture set continues to be necessary, due to its practical utility in research and emotional assessment. In any case, and in the line of Botdorf et al. (2017), evidence supports the use of this instrument by researchers who study cognitive control in the laboratory, where it can evoke an affective state to adequately explain risk decisions in adolescence.

As for the statistically significant differences found between groups, results indicate that these occur primarily between the group of secondary school students and the group of adolescents under custodial sanctions. Specifically, statistically significant differences were found in valence dimension in the assessment of unpleasant pictures, indicating that the custody group perceived this type of picture higher in pleasantness. In this case, the group with the least severity of antisocial behavior (non-custodial) is equated with the comparison group. Serafim et al. (2009), who assessed heart rate in a group of antisocial adult psychopaths, a group of non-psychopathic adults with an antisocial career, and a comparison group of adults, found differences in this variable when subjects observed different emotionally charged IAPS pictures. Their results indicated that the group of psychopaths showed scarcely any variation in heart rate under the different stimuli, while the comparison group showed the greatest variation, and the antisocial, non-psychopath group fell somewhere in the middle. These results suggest that heart rate, a physiological response that is related to the valence dimension (Bradley and Lang, 2007), could be different between the antisocial groups (psychopaths or not) and the comparison group, in response to different emotional stimuli, in agreement with our own results. Therefore, these data once again point to the existence of a deficit or impairment in the emotional processing capacity of antisocial populations, mainly affecting stimuli with a negative valence. This fact could have implications in the study and comprehension of other cognitive processes and functions, since a variety of evidence points to the influence of unpleasant stimuli on capacities of long-term memory (Fernández-Rey and Redondo, 2007) or recognition memory (Gordillo-León et al., 2010) or specifically in children and adolescents with antisocial behavior, who show reduced startle reflex to aversive pictures (Fairchild et al., 2010), moreover, the magnitude of the startle and skin conductance responses evoked by the emotional stimuli was inversely related to the severity of the antisocial behavior (Syngelaki et al., 2013). Statistically significant differences were found in arousal dimension in the assessment of unpleasant pictures, indicating that the custody group perceived this type of picture with more arousal higher in pleasantness.

In addition, differences are found in the arousal dimension in the evaluation of all images, indicating, that the custody group has greater activation than the non-custody and student groups, in the perception of pleasant and neutral images. This result indicates the impulsivity component associated with young people with antisocial behavior (Rockstroh and McTeague, 2019; Toro et al., 2020) which is consistent with a recent study finding an association between higher ratings of arousal to positive images and attention deficit hyperactivity disorder symptoms in youth (Truedsson et al., 2019). Furthermore, the results showed that custody sample were associated with lower ratings of arousal to unpleasant images, and concurrently in line with previous finding with CU in youth samples (Truedsson et al., 2019; Szabó et al., 2020).

On the other hand, the results obtained in the discriminant analyses of the three affective dimensions support the differences found between the groups according to picture type presented. These analyses indicate, in each dimension, that there are two main functions, however, greater between-group variance is always associated with the first function. These first functions draw together the unpleasant pictures with violent and/or aggressive content (gun, knife, masked man, mugging, or robberies on trains and subways), in every dimension. The custody group is furthest from the group of students in normal schooling, also in all three dimensions, and the group of antisocial adolescents under non-custodial judicial measures falls in the middle. Moreover, given that adolescents show greater sensitivity to motivational and socio-emotional information, they are potentially more vulnerable than older youths and adults to bad decision making in these situations (Cohen-Gilbert and Thomas, 2013). For these reasons, future research and programs designed to curb risk decisions may benefit from focusing on ways to improve adolescent cognitive control, specifically in negatively charged situations in antisocial contexts (Botdorf et al., 2017). The executive processes and cortical networks associated with EF functioning suggest the existence of two differentiated circuits: the executive control circuit (dorso-lateral), involved in cold, more cognitive EFs, and the socio-emotional circuit (orbito-frontal and ventro-medial), involved in hot processes related to behavioral and emotional control (Zelazo et al., 2004; Zelazo, 2020). EFs play an important role in socio-emotional circuit involved in hot processes, so we join Zelazo (2020) in underscoring the importance of working on EF development as a protective factor against possible difficulties in emotional development, especially in the stage of adolescence.

Finally, some limitations of the study should be pointed out: in particular, a larger sample would be needed to continue providing evidence of the validity of the IAPS in Spanish adolescents with antisocial behavior and in normalized groups. In addition, no alternative measure of the emotional state of adolescents or even psychophysiological measures have been considered.

## Data Availability Statement

The raw data supporting the conclusions of this article will be made available by the authors, without undue reservation.

## Ethics Statement

The studies involving human participants were reviewed and approved by the University Bioethics Commission of Research with Humans, University of Almeria (ref. 170214). Written informed consent to participate in this study was provided by the participants' legal guardian/next of kin.

## Author Contributions

All authors contributed in all the phases of the research and the elaboration of the article. The authors have read and agreed to the published version of the manuscript.

## Conflict of Interest

The authors declare that the research was conducted in the absence of any commercial or financial relationships that could be construed as a potential conflict of interest.
